# Case report: Intranasal esketamine for severe major depressive disorder with psychotic features

**DOI:** 10.3389/fpsyt.2022.937996

**Published:** 2022-07-25

**Authors:** Maximilian Carter, Kassandra Solsrud, Nicholas Mischel

**Affiliations:** Department of Psychiatry and Behavioral Neurosciences, Wayne State University School of Medicine, Detroit, MI, United States

**Keywords:** esketamine, depression, psychotic, case report, intranasal

## Abstract

**Introduction:**

About one third of patients with major depressive disorder (MDD) have treatment resistant depression (TRD). The difficulty of treating TRD especially in those with suicidal ideation and psychotic features demands treatments that are fast-acting, safe, and effective. Limited access, lack of viable options, and incomplete characterization of rapid-acting antidepressants has prevented widespread incorporation into treatment of patients with TRD. However, ketamine and its variations have shown promise of being effective treatment options for patients with TRD with psychotic features.

**Case description:**

This 28-year-old patient with TRD with psychotic features received 14 treatments of intranasal esketamine over a 3-month period. This patient initially presented with anhedonia, difficulty sleeping, suicidal thoughts, and auditory hallucinations. The Quick Inventory of Depressive Symptomology (QIDS) was used to assess depression before each session.

**Results:**

After her first two treatment sessions within a week, this patient experienced a reduction in depression from severe to moderate according to the QIDS. Over 14 sessions, she had no significant adverse effects, including no psychotic symptoms during esketamine treatment, and was stabilized to mild depression without suicidal ideations. One year after treatment, she continues to be stable. She has not had auditory hallucinations since the esketamine treatment.

**Conclusions:**

This case report provides an example of a patient with severe TRD with psychotic features that showed significant improvement after treatment with intranasal esketamine. Larger studies are indicated to further elucidate the effectiveness and safety of intranasal esketamine, so it can be more widely used for patients with TRD with psychotic features.

## Introduction

Over 350 million people in the world suffer from depression (1, 2). Since the COVID-19 pandemic began, there has been an increase in the number of people with depression, with some estimates showing a 27.6% increase per 100,000 (2). About one third of patients with depression have treatment resistant depression (TRD) (1). TRD is considered to be failure to respond to two antidepressant monotherapies (3). The delayed onset of action of antidepressants leaves patients with TRD at risk for suicidal behavior (4). Finding treatments with shorter onsets of action is essential to better patient care.

Ketamine, an NMDA receptor antagonist, has been shown to have a rapid-acting antidepressant effect that can reduce suicidal thoughts in 1 day (1, 2, 4). The US FDA approved the use of intranasal esketamine for TRD in 2019 (5). Multiple randomized control trials have demonstrated the effectiveness of intravenous ketamine as an antidepressant (3, 6). However, intravenous infusions are not a convenient administration route and can be resource intensive. Alternate administration options have been explored including intranasal ketamine, which has been shown to be a viable alternative (3, 6). Because esketamine, the S-enantiomer of ketamine, is much more potent than ketamine, it can be used at lower doses (1, 4, 5).

The effectiveness and safety of esketamine in the treatment of patient with TRD has not been fully characterized. Due to ketamine's cardiovascular effects including elevations in heart rate and blood pressure, it is important to monitor vital signs during administration (6). Adverse effects, such as dissociative symptoms, have been observed at higher doses (1). It has been previously thought that patients with a primary psychotic disorder or MDD with psychosis should not be started on esketamine due to the potential worsening of the psychotic features. Yet, there has been very limited research into the use of ketamine in TRD patients with psychotic features (7). Some preliminary findings suggests that ketamine treatment for patients with depression with psychotic features is effective and well-tolerated (7). Subcutaneous esketamine treatment for TRD with psychotic features has been found to be safe and effective in small studies (8). However, there has been no research on the use of intranasal esketamine in TRD patients with psychosis. Because ketamine has only been approved for a short period of time, there is still a need to explore how effective and safe it is as a treatment for TRD. There is insufficient data on the effectiveness of ketamine vs. esketamine. In this case report, we discuss a patient with severe MDD with psychotic features that was treated with intranasal esketamine.

## Case description

The patient is a 28-year-old African American female with severe MDD with psychotic features and previously diagnosed generalized anxiety disorder (GAD). Her medical history includes essential hypertension and asthma. She has no known family history of mood or psychotic disorders. Several days before her scheduled initiation of esketamine treatment, she presented to the hospital for thoughts about cutting her wrists and cutting her throat with a knife. She was admitted to the crisis center for 3 days.

Before treatment, she had frequent, intrusive thoughts of harming herself and others. She admitted to vivid visual hallucinations of stabbing herself with a knife. Before treatment, she had increasingly frequent episodes of watching herself harm others through her visual hallucinations. The patient admitted auditory hallucinations of being criticized by herself and others. She heard her own voice telling her that she “was not good enough.” She heard voices of friends and family telling her the same thing. She also endorsed hearing voices with which she was not familiar. Patient denies previous suicide attempts or a specific plan.

## Diagnostic assessment

This patient's symptoms initially included excessive worrying, difficulty sleeping, increased fatigue, and muscle aches. She was diagnosed with anxiety and prescribed Escitalopram. In her mid-twenties, she presented to the emergency department several times for suicidal behavior and the diagnosis of depression was added. Laboratory studies evaluating for endocrine diseases, vitamin deficiencies, and infections were negative for causes of secondary depression. Prior to 2020, she did not receive consistent psychiatric care, thus she was never properly evaluated, diagnosed, or treated. Previous treatment included trials of Desvenlafaxine, Escitalopram, and Nortriptyline without successful reduction in symptoms. In 2020, she presented to the emergency department for hearing voices. Psychotic disorders, such as schizoaffective disorder, and bipolar depression with psychotic features were considered, but her primary symptoms pointed toward a mood disorder. The patient had no manic or hypomanic episodes and her psychotic symptoms occurred during mood episodes. Her symptoms of depressed mood, anhedonia, insomnia, fatigue, and suicidal thoughts in combination with auditory hallucinations best fit a diagnosis of MDD with psychotic features over a primary psychotic disorder.

A range of pharmaceutical and non-pharmaceutical treatment options were considered, including electroconvulsive therapy, transcranial magnetic stimulation, cognitive behavioral therapy (CBT), alternate second-generation antipsychotics, and alternate mood stabilizers. The patient had already tried CBT in combination with a number of antidepressants, antipsychotics, and mood stabilizers without adequate response. Therefore, it was decided that esketamine could be an effective treatment for this patient. Before being started on intranasal esketamine, she was on Bupropion 150 mg, Aripiprazole 10 mg, Trazodone 100 mg, Clonazepam 0.5 mg, and Sertraline 50 mg.

The patient had a total of fourteen treatment sessions with esketamine over a period of 3 months. We followed the FDA labeled treatment protocol in which doses are fixed at 56 mg for induction and 84 mg thereafter. The patient was informed of the benefits and risks of this treatment and agreed to the course. The first eight sessions occurred biweekly, then four sessions weekly, then two session bimonthly. This regimen was chosen to ensure that the esketamine was safely titrated and tapered. The Quick Inventory of Depressive Symptomology (QIDS) was used to evaluate depressive symptoms. The QIDS was completed at the beginning of each session. The treatment sessions were followed by vital sign monitoring for 2 h and assessment of changes in psychotic symptoms by one-on-one monitoring with a therapist. The patient did not experience any psychotic symptoms during the treatment sessions. She did endorse mild nausea and headache during one of the sessions, but the patient did not experience any significant adverse effects. Even though the patient has comorbid hypertension, she did not experience any significant vital sign changes during the treatment sessions.

**Figure 1:**
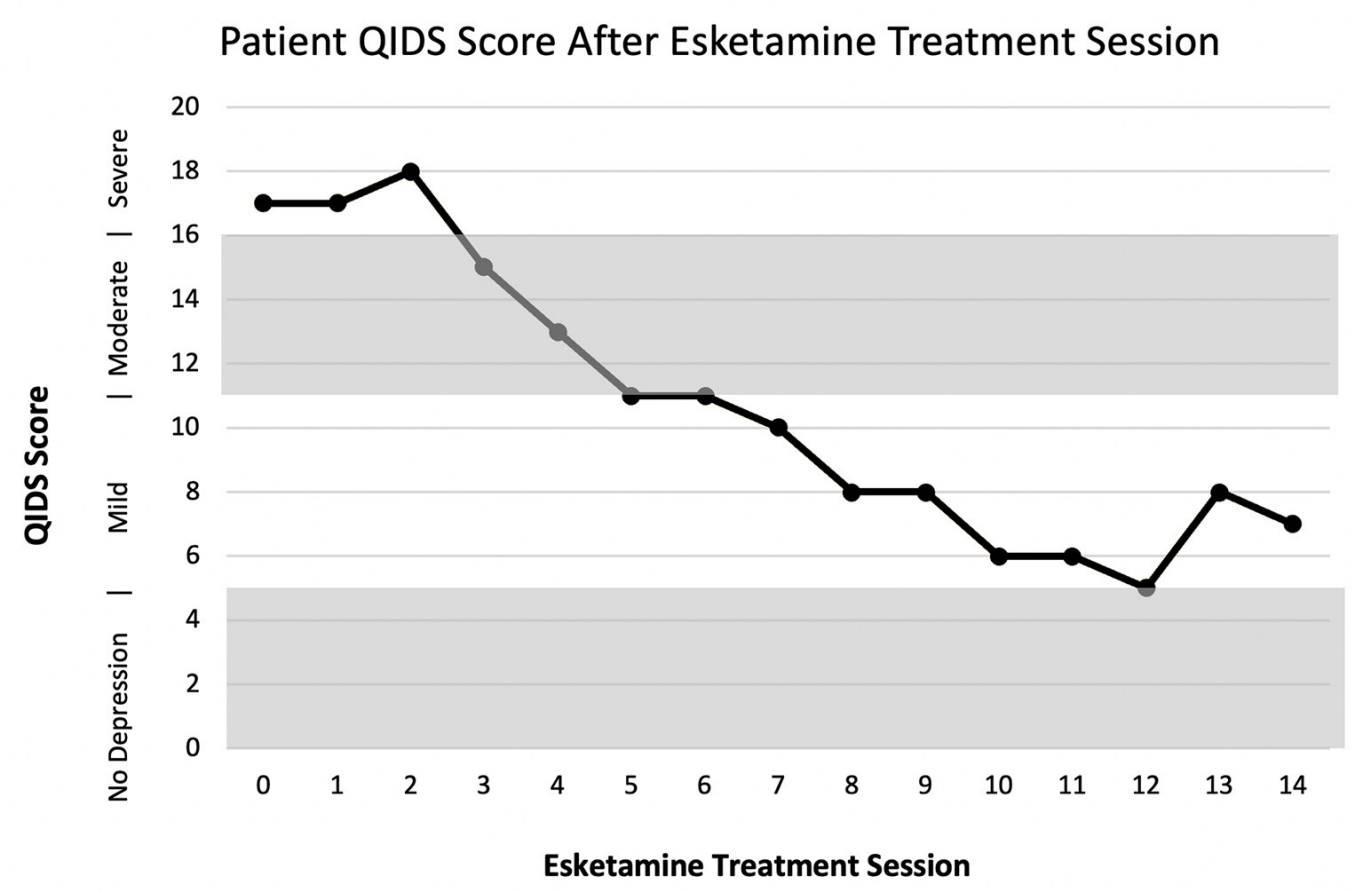
Patient QIDS score after treatment with Esketamine.

Before treatment the patient had a QIDS score of 17, which indicates severe depression (Figure 1). The first two sessions the patient received an esketamine dose of 56 mg without side effects. The subsequent doses were increased to 84 mg and were well-tolerated. One week into treatment at the third session, the patient had a reduction in QIDS to 15, which indicates moderate depression. At her seventh treatment, 3 weeks after initiation of esketamine, she had a further reduction in QIDS to 10, indicating mild depression. The patient continued to show improvement with a decrease in QIDS to 5 at treatment session twelve. There was an increase to eight at treatment session thirteen at which the patient reported difficulty sleeping and having a headache. Nine months after completing treatment, she is stable with a QIDS of eight after discontinuing Bupropion and continuing Aripiprazole 10 mg, Trazodone 100 mg, Clonazepam 0.5 mg, and Sertraline 50 mg.

Since treatment, the patient no longer hears voices. She has had significantly fewer episodes of the visualizations of self-harm. When she has these thoughts, she says that they are not as vivid as before the esketamine treatment and they only occur during periods of stress. She denies any thoughts of harming others.

## Discussion

There is growing evidence that ketamine is a viable treatment option for patients with MDD with psychotic features (1, 7, 8). Ketamine treatments can have a quicker onset of action and be used after other treatments have failed. Thus, it is important to understand the safety profile of the various ketamine medications and administration routes to quickly and effective help patients with TRD, especially those at risk for suicidal or homicidal behavior.

We describe a patient with severe TRD with psychotic features that was successfully treated with intranasal esketamine. Before treatment the patient was admitted to the hospital multiple times for suicidal thoughts and auditory hallucinations. She was diagnosed with severe MDD with psychotic features. This patient showed a reduction in depressive symptoms and suicidal thoughts after only two intranasal esketamine sessions. After intranasal esketamine treatment completion, the patient denied having any auditory hallucinations and has had a significant reduction in the intrusive thoughts of self-harm. The 14 treatment sessions resulted in an overall improvement in her depression to mild according to QIDS. The intranasal esketamine treatment was well-tolerated. It is important to do safety monitoring for patients during intranasal esketamine administration, especially those with psychotic features, and assess for changes in psychotic symptoms. Effectively treating patients with TRD can be difficult, and it is further complicated because there is a reluctance to use ketamine and its derivatives in patients with psychotic symptoms due to adverse effects. However, this case demonstrates that appropriate dosing of esketamine along with careful monitoring can be an effective therapy without eliciting psychotic symptoms during treatment. Further research is warranted to better understand this valuable treatment. For partial recurrence of similar symptoms, we would pursue a shorter series of treatments. For a full recurrence, we would likely plan a repeat course. Should the durability of esketamine not be reasonably sufficient (6 months or more), we would recommend revisiting treatment options. Ultimately our findings support the limited data that suggest that ketamine is a safe and effective treatment for patients with MDD with psychotic features (7, 8).

It is important to do more research to determine the long-term effects of intranasal esketamine treatment, if longer treatment courses would completely reduce depressive symptoms, and if maintenance treatments are required. There are limitations to this study, including the lack of a control. Because this case report only describes one patient, the results here cannot be generalized to the larger population without further investigation. The safety and effectiveness of intranasal esketamine in patients with MDD with psychotic features requires larger studies.

## Patient perspective

The patient said that the intranasal esketamine treatment helped her by reducing her suicidal thoughts and allowed her to feel less depressed. She admitted to having nausea and headache during one of the sessions but did not have other side effects. Before treatment, she felt like she had pain in her body but had difficulty finding where the pain was coming from. Sometimes this pain manifested as a headache. After treatment, she says that she can now sit down and think about where the pain is coming from, and it goes away. She says that she is more in tune with how her body is feeling. She has less anhedonia and feels less anxious. She has not had to miss work as often and is able to work more effectively. She is able to spend more time doing things that she enjoys, such as taking care of and playing with her children. She admits that the treatment allowed her to understand reality better. She can now differentiate reality from her hallucinations. Overall, she feels that the treatment was very beneficial and improved her quality of life.

## Conclusion

This case report provides an important example of intranasal esketamine being used effectively and safely to treat a patient with severe TRD with psychotic features. The treatment was well-tolerated as the patient did not experience significant side effects. While these results cannot be generalized, this is a promising case of how intranasal esketamine is a rapid-acting treatment that can reduce suicidal thoughts in patients with MDD with psychotic features. There is a need for more in-depth and larger analysis to further investigate the efficacy and safety of intranasal ketamine for patients with MDD with psychotic features.

## Data availability statement

The raw data supporting the conclusions of this article will be made available by the authors, without undue reservation.

## Ethics statement

Ethical review and approval was not required for the study on human participants in accordance with the local legislation and institutional requirements. The patients/participants provided their written informed consent to participate in this study. Written informed consent was obtained from the individual(s) for the publication of any potentially identifiable images or data included in this article.

## Author contributions

MC: conceptualization, data collection and analysis, writing, and editing. KS: data analysis, writing, and editing. NM: conceptualization, editing, and supervision. All authors contributed to the article and approved the submitted version.

## Conflict of interest

The authors declare that the research was conducted in the absence of any commercial or financial relationships that could be construed as a potential conflict of interest.

## Publisher's note

All claims expressed in this article are solely those of the authors and do not necessarily represent those of their affiliated organizations, or those of the publisher, the editors and the reviewers. Any product that may be evaluated in this article, or claim that may be made by its manufacturer, is not guaranteed or endorsed by the publisher.
